# Milk microbial composition of Brazilian dairy cows entering the dry period and genomic comparison between *Staphylococcus aureus* strains susceptible to the bacteriophage vB_SauM-UFV_DC4

**DOI:** 10.1038/s41598-020-62499-6

**Published:** 2020-03-26

**Authors:** Vinícius da Silva Duarte, Laura Treu, Cristina Sartori, Roberto Sousa Dias, Isabela da Silva Paes, Marcella Silva Vieira, Gabriele Rocha Santana, Marcos Inácio Marcondes, Alessio Giacomini, Viviana Corich, Stefano Campanaro, Cynthia Canedo da Silva, Sérgio Oliveira de Paula

**Affiliations:** 1 Department of Microbiology, Federal University of Viçosa, Av. Peter Henry Rolfs, s/n, Campus Universitário, 36570-900 Viçosa, Minas Gerais Brazil; 2 Department of Biology, University of Padova, Via U. Bassi 58/b, 35121 Padova, Italy; 3 Department of Agronomy Food Natural Resources Animals and Environment, University of Padova, Viale dell’Universitá, 16, 35020 Legnaro, PD Italy; 4 Department of General Biology, Federal University of Viçosa, Av. Peter Henry Rolfs, s/n, Campus Universitário, 36570-900 Viçosa, Minas Gerais Brazil; 5 Department of Animal Science, Universidade Federal de Viçosa, Viçosa, Brazil; 6 CRIBI Biotechnology Center Viale G. Colombo 3, 35121 Padova, Italy

**Keywords:** Applied microbiology, Bacteriophages, Clinical microbiology

## Abstract

Brazil has the second-largest dairy cattle herd in the world, and bovine mastitis still can cause significant losses for dairy farmers. Despite this fact, little information is available about milk microbial composition of Brazilian dairy cows, as well as the potential use of bacteriophages in the control of *S. aureus*. Here, we investigated milk bacterial composition of 28 Holstein Fresian cows (109 teats), selected in the dry-off period, using 16S rRNA analysis. Furthermore, a representative *S. aureus* strain (UFV2030RH1) was obtained at drying-off for isolation of a bacteriophage (vB_SauM-UFV_DC4, UFV_DC4) and bacterial genomic comparison purposes. Our outcomes revealed that *Staphylococcus* was the third most prevalent genus and positively correlated with subclinical mastitis events. As a major finding, genomic analyses showed the presence of adhesive matrix molecules that recognize microbial surface components (MSCRAMM) in UFV2030RH1 and might indicate great biofilm formation capability. A minimum inhibitory concentration (MIC) assay showed that resistance to ampicillin was the highest among the antibiotic tested in *S. aureus* 3059 and UFV2030RH1, displaying values four and sixteen times greater than MIC resistance breakpoint, respectively. Together, our results suggest that *Staphylococcus* is highly prevalent in dairy cows at drying-off and the use of the phage UFV_DC4 as a biocontrol agent must be investigated in future studies.

## Introduction

In the last few years, dairy activity in Brazil has undergone deep changes, focusing mainly on the improvement of the productive lifetime of dairy cows (i.e. milk production from birth to culling) and the possibility to reduce operation costs. Nowadays, Brazil is the fifth largest global milk producer and it has the second-largest dairy herd in the world, reflecting its relevance to the international dairy market^1^.

Overall, the cornerstones of a successful dairy farm activity are related to well-established nutritional, genetic and sanitary managements. As regards the latter, bovine mastitis is still the leading cause of economic losses for dairy farmers with an estimated economic impact of US$ 91,500 annually, based on a case study involving 142 lactating Holstein cows under tropical conditions in Minas Gerais, Brazil^2^.

Bovine mastitis is considered a multifactorial disease, where microbiological agents (mainly bacteria), the environment and intrinsic cow factors must be taken into consideration for treatment choice^3^. Currently, high-throughput sequencing (HTS) technology, such as 16S rRNA analysis and whole metagenome sequencing (WMS), allows an accurate and in-depth evaluation of milk microbiome profile useful for therapeutic purposes ^4–7^. However, little or no information is available about milk microbial composition in Brazilian dairy cows in any lactation period that has been assessed by using HTS technologies. With the application of culture-dependent techniques, more than 150 bacterial species have been isolated from udders with mastitis, which reflects the vast majority of mastitis cases having bacterial etiology^8^. *Staphylococcus aureus* remains the main etiological agent in cases of contagious mastitis (40–70%), whereas *Escherichia coli* accounts for the majority of cases of environmental mastitis (40%).

The routine monitoring of subclinical mastitis in Brazilian farms is mainly performed through the California Mastitis Test (CMT) and Somaticell^®^, both cow-side tests considered as practical, inexpensive, user-friendly and providing quick results. Treatment of bovine mastitis is typically based on the use of short and long-acting antibiotics during the lactation and dry-off period, respectively^8,9^.

With respect to the dry cow therapy (DCT), its adoption is part of udder health management practices to prevent the occurrence of new intramammary infection (IMI) before the upcoming lactation^10^. Historically, blanket dry cow therapy (BDCT) has been adopted as strategy to treat all cows with intramammary antimicrobials at dry-off period, albeit some European countries have chosen the use of selective antibiotics (selective dry cow therapy, SDCT) to reduce antibiotic use in livestock species, in which animal identification and treatment are based on criteria such as somatic cell count (SCC), bacteriological culture, clinical mastitis history and antibiotic use^10,11^. Despite BDCT or SDCT being routinely adopted among dairy farmers, both practices are currently considered as questionable for use in healthy herds due to their effectiveness and the emergence and spread of antimicrobial resistance (AMR)^4,10^.

In this scenario, bacterial viruses (phages or bacteriophages), or more recently virion-associated peptidoglycan hydrolases (VAPGHs), arise as a potential tool against antibiotic-resistant bacteria in dairy cows, as demonstrated by several studies^12–16^. Specifically regarding *Staphylococci* phages, the vast majority are temperate siphoviruses^17^. Lytic bacteriophages of the *Myoviridae* family targeting *S. aureus* are attracting special attention owing to their broad host range and promising perspective in terms of phage therapy^18^. Several studies have been published on staphylococcal phages showing different isolation sources (e.g. sewage systems, ponds, soil samples and human tissues), genomic features and their effectiveness *in vitro* and *in vivo*^18–20^; however there are only a few numbers of studies in the literature on bacteriophages isolated for pathogens associated with IMI on the dry-off period.

In view of the potential use of phages for therapeutic purposes and the current understanding of how bacteriophages can impact bacterial communities^21,22^, we evaluated the milk microbial composition at dry-off by means of a culture-independent approach focusing on the relative abundance of the genus *Staphylococcus*. Moreover, *S. aureus* UFV2030RH1 was isolated from a dairy cow at drying-off and used to isolate the bacteriophage vB_SauM-UFV_DC4 existing on the wastewater of a dairy farm. Finally, the *S. aureus* UFV2030RH1 genome was sequenced and compared with one originated from a clinical *S. aureus* strain causing mastitis.

## Materials and methods

### Animals, housing and milk samples collection

We collected the milk from each mammary gland quarter, named right hind (RH), right front (RF), left hind (LH) and left front (LF), from twenty-eight Holstein cows (primiparous n = 20; multiparous n = 8). The animals were housed in a mattress-bedding free stall with sawdust over the beds, replaced twice a day. Only dairy cows that have been chosen to enter the dry-off period from October 2015 to July 2016 in a single dairy farm belonging to the Departamento de Zootecnia, Universidade Federal de Viçosa (DZO/UFV) were enrolled in this study. All individual cows history such as age, breed, number of lactations, CMT scores (0 to 3), clinical mastitis events and antibiotic of choice for treatment were considered (Supplementary Spreadsheet S1) and used for statistical analysis (item 2.7).

Prior to milking, teats were cleaned (pre-dipping) with a chlorine solution (750 ppm), dried with disposable towels and the first three milk jets discarded on a “strip cup” for each mammary gland quarter. Milk sampling was performed by manual milking into sterilized 15 mL polyethylene sterile tubes in the afternoon of the dry-off day. The sampler wore disposable gloves. Afterward, the animal assigned for dry off received an intramammary infusion of 600 mg of benzathine cloxacillin (Orbenim^®^ Extra Dry Cow, Zoetis Inc. Kalamazoo) or 250 mg of Cephalonium (Cepravim^®^ Dry Cow, MSD Animal Health) depending on its clinical or subclinical mastitis history (Supplementary Spreadsheet S1).

Samples were kept on ice and transported to Laboratório de Imunovirologia Molecular at Universidade Federal de Viçosa (LIMV/UFV), Viçosa, Minas Gerais, Brazil, for bacterial isolation and stored at −20 °C for further molecular analyses.

It is worth mentioning that milk collection and animal management occurred according to the daily routine procedures adopted in this dairy farm. Therefore, an evaluation from the Ethics Committee for Animal Use was not necessary for this study. The authors declare that they followed the rules on the use of animals for research of the Universidade Federal de Viçosa (Comissão de ética no uso de animais/CEUA) as described in Section II, Art. 6°.

### *S. aureus* strains and culture conditions

*S. aureus* UFV2030RH1 was isolated from the mammary gland quarter RH of the animal identified as 2030, enrolled in this study. and it was used as a host for viral isolation. Briefly, a milk sample was streaked in Brain Heart Infusion (BHI) agar and incubated at 37 °C for 24 h. Golden colonies were picked up and restreaked onto BHI agar and, after incubation (37 °C, 24 h), subjected to Gram staining and catalase test. The strain *S. aureus* 3059, also used as a host for viral isolation, was isolated from dairy cows with clinical mastitis and kindly provided by EMBRAPA Dairy Cattle (Juiz de Fora, Minas Gerais, Brazil). Both strains were routinely cultured using BHI liquid broth or agar at 37 °C for 24 h.

### Isolation and morphological characterization of UFV_DC4

Wastewater is considered a good source for screening phages against *S. aureus* because of its high viral content^23^. Therefore, samples from wastewater of a dairy farm located at the Departamento de Zootecnia, Universidade Federal de Viçosa (DZO/UFV) at Viçosa, Minas Gerais, Brazil, were collected and used for viral isolation. Samples were kept on ice until centrifugation (12,000 *g* at 4 °C for 15 min). After dilution (1:4) in SM buffer (5.8 g/L NaCl, 2.0 g MgSO4.7H2O, 50 ml Tris-HCl 1 M, 5 ml gelatin 2%, pH 7.5), viral suspension was double-filtered through pore-size PES membranes (0.45 and 0.22 µm) (Millipore, Billerica, MA, USA) and added to an early log-phase (O.D_600 nm_ of 0.3) cultures of *S. aureus* UFV2030RH1 grown in BHI broth. The mixture was incubated at 37 °C for phage attachment and then plated using the standard soft agar overlay technique. Plates were incubated overnight at 37 °C. Each lysis plate was recovered and propagated three times to guarantee that a unique bacteriophage was isolated.

For transmission electron microscopy (TEM), phage particles were purified using sucrose cushion as described by Bourdin *et al*.^24^. TEM was conducted at Núcleo de Microscopia e Microanálise (NMM) at UFV. A 10 µL aliquot of the viral suspension was deposited on FormVar-coated grids with 200 meshes and negatively stained with 2% uranyl acetate. Samples were then visualized using a Zeiss EM 109 electron microscopy operating at 80 kV. Images were analyzed using ImageJ^25^. The virus UFV_DC4 was classified according to criteria established by the International Committee on Taxonomy of Viruses (ICTV)^26^.

### Antimicrobial susceptibility test

Antimicrobial susceptibility tests on *S. aureus* 3059 and UFV2030RH1 were done by the disc diffusion assay. In total, a set of 25 different antibiotics (Supplementary Table S1) (DME Polisensidisc, Araçatuba, São Paulo, Brazil) was used and the assay conducted according to the manufacturer’s recommendation. The results were interpreted according to the Clinical and Laboratory Standards Institute (CLSI)^27^.

The minimum inhibitory concentration (MIC) assay was performed on 96-well microtiter plates (broth microdilution methodology) using the antibiotics used for drying the animals (i.e. cephalonium, ceftiofur and ampicillin), as described by Wiegand *et al*.^28^. In brief, the inoculum was prepared by growing each bacterial strain in Muller-Hinton (MH) broth for 24 h and then adjusting the O.D_600 nm_ to 0.1 (10^7^ CFU/mL) with sterile medium. This was further diluted to the final concentration of 5 × 10^5^ CFU/mL in each well. Each antimicrobial tested was diluted (1:1) and wells contained twelve different concentrations (128 to 0.25 µg/mL). This assay was carried out in triplicate.

### DNA extraction and sequencing

#### Milk samples at dry-off

Bacterial DNA from milk at drying off was extracted by adapting the protocol described by Stevenson and Weimer^29^. Briefly, Frozen milk samples (15 mL) were thawed at 4 °C and centrifuged at 5,000 *g* for 25 min at 4 °C. Cell pellet was resuspended with 2 mL of extraction buffer (100 mM Tris-HCl, 10 mM EDTA, 0.15 M NaCl, pH 8.0) and transferred to screw-cap 2.0 mL microfuge tube containing 0.5 g of 0.1 mm diameter zirconium beads. An aliquot of 50 μl of cold 20% SDS and 700 μl of equilibrated phenol (pH 8.0) were added and a mechanical lysis procedure was applied (bead-beat apparatus, 2 min, 4 °C). After incubation at 60 °C for 10 min, mechanical lysis was performed again using the same conditions described above, then samples were centrifuged at 12,000 *g* for 10 min, and finally the aqueous phase was transferred to new tube. After a phenol: chloroform (1:1) and chloroform steps (12,000 *g* for 5 min at 4 °C), the supernatant was transferred to new microfuge tubes and 1/10 volume of 3 M sodium acetate and 0.6 volume of isopropanol were added. Centrifugation at 12,000 *g* for 20 min at 4 °C was conducted and the DNA washed using 70% and 100% ethanol (12,000 *g* for 5 min at 4 °C). Finally, DNA was dried at room temperature for 40 min and dissolved in 50 μL of sterile deionized water.

The DNA concentration was determined using a NanoDrop (NanoDrop 2000c, Thermo Scientific, USA) and the samples were lyophilized. The V4 region of the 16S rRNA gene was amplified by PCR using 515 f/806r primers and amplicons sequenced using Illumina MiSeq desktop sequencer (Argonne National Laboratory, Illinois, USA) producing 150 bp paired-end (PE) reads. According to previous verifications^30^, the number of reads generated is sufficient to identify the main taxonomic groups present in the samples (Supplementary Fig. S1; Supplementary Spreadsheet S1 – rarefaction curves).

#### *S. aureus* 3059 and UFV2030RH1

Bacterial DNA extraction was performed using the protocol described by Pospiech and Neumann^31^. Bacterial cells were grown in BHI broth until stationary phase (O.D._600 nm_ of 0.7), harvested by centrifugation (4,500 *g* for 5 min at 4 °C) and resuspended in 5 mL of SET buffer (75 mM NaCl, 25 mM EDTA, 20 mM Tris, pH 7.5). Afterward, lysozyme (1 mg/mL) was added to the bacterial cell suspension followed by incubation at 37 °C for 60 min. After this period, 1/10 volume of 10% SDS and 0.5 mg/mL proteinase K was added and incubated at 55 °C for 2 h. Then 1/3 volume of 5 M NaCl and 1 volume of chloroform were added and incubated at room temperature for 30 min with frequent gentle tube inversion. After centrifugation (4,500 *g* for 15 min), the aqueous phase was carefully collected, transferred to a new tube containing 1 volume of isopropanol, mixed and centrifuged at 12,000 *g* for 15 min at 4 °C. DNA pellet was washed with room-temperature 70% ethanol and after air-drying for 20 min, DNA was resuspended in 100 µL of nuclease-free water.

The purity and quality of the DNA were checked by electrophoresis on 0.8% agarose gel and DNA concentration was estimated by measuring the absorbance at 260 nm using a NanoDrop 2000 (Thermo Fisher, USA). Genomic bacterial DNA was sent to the Molecular Research DNA (Shallowater, TX, USA; mrdna.com). The Nextera XT DNA Library Preparation Kit (Illumina Inc, San Diego, CA, USA) was used for generation of the genomic libraries and whole-genome sequencing was performed with the Illumina MiSeq platform using PE reads (2 ×150 bp).

### Bioinformatics analyses

#### Bacterial composition using the 16S rRNA region

The open-source pipeline Quantitative Insights into Microbial Ecology (QIIME 1.9.1+dfsg)^32^ was used to process 109 independent 16S rRNA sequenced samples. Raw reads were trimmed, quality-filtered using Trimmomatic^33^ and chimeric sequences removed using USEARCH^34^. Operational taxonomic units (OTUs) were picked against the GreenGenes 13_5 database at a threshold of 97% similarity. Sequences derived from mitochondrial DNA were identified and removed after checking similarity to a database and using Bowtie 2 for alignment^35^. The alignment was performed using standard parameters except than “–very sensitive local”; unmapped reads were sorted with SAMtools^36^ and saved in a separate file. OTUs representative sequences were compared with the 16S ribosomal RNA sequences database with MegaBLAST (ncbi.nlm.nih.gov/BLAST/). OTUs abundance of each animal quarter, considered as the relative abundance of the 95% most abundant OTUs, was hierarchically clustered using the average linkage method with Euclidean Distance as the distance metric and represented using a heat map created with MultiExperiment Viewer (MeV 4.9.0)^37^. Principal component analysis (PCA) was performed to assess the similarity between microbial communities using STAMP software^38^.

#### *S. aureus* strains

Bacterial sequences were assembled *de novo* using the CLC Genomics Workbench software (version 9.5). *S. aureus* UFV2030RH1 and 3059 scaffolds were ordered and oriented, respectively, using *S. aureus* ATCC 6538 and NCTC10344 as the reference genomes with MeDuSa^39^. The Rapid Annotation using Subsystem Technology (RAST server)^40^ was used for gene annotation. Furthermore, protein-coding genes from each bacterial genome were functionally annotated using GhostKOALA^41^. The PHAge Search Tool (PHASTER) webserver^42^ was used to predict prophage regions, while arrays of clustered regularly interspaced short palindromic repeats (CRISPR) were searched using the algorithm CRISPRDetect^43^. CRISPR arrays were visualized with CRISPRStudio enabling default parameters^44^. Unique spacers were checked against the PHAST database^45^. *In silico* detection of antibiotic resistance genes was conducted using the Comprehensive Antibiotic Resistance Database (CARD)^46^.

For comparative analysis, we downloaded 27 *S. aureus* whole-genome sequences available at NCBI GenBank considered as a reference or associated with subclinical manifestations of mastitis (Supplementary Table S2). A fragmented all-against-all comparison in TBLASTX mode was conducted with Gegenees software^47^, setting the parameters to 500/500 (frag-size/slide-size). The heat plot was generated setting a maximum threshold (40%) in order to obtain the best phylogenomic overview. An unrooted phylogenetic tree was computed using SplitsTree4^48^.

#### 16S rRNA raw reads deposit and *S. aureus* genomes accession numbers

Raw reads were deposited in the Sequence Read Archive (SRA) database (http://www.ncbi.nlm. nih.gov/sra) under the BioProject PRJNA437620.

The whole-genome shotgun project of *S. aureus* UFV2030RH1 and 3059 have been deposited at DDBJ/ENA/GenBank under the accession numbers CP039848 and SSWQ00000000, respectively; the versions described in this paper are CP039848 and SSWQ01000000, respectively for *S. aureus* UFV2030RH1 and 3059.

### Statistical analysis

We have focused on the temporal window from February 2015 to July 2016. The whole lactation for each cow occurred in that period and was periodically sampled (about one sampling per month, on average 14.55 ± 2.78 samplings per cow). Milk sample from each quarter was systematically subjected to the California Mastitis Test (CMT) for the occurrence of subclinical mastitis events, scored from 0 (absence) to 3. The occurrence of clinical mastitis and its treatment were also annotated (Supplementary Spreadsheet S1).

As measures of microbial diversity at drying off, the Shannon diversity index was calculated using PAST (version 3.20)^49^. Moreover, in order to investigate the effect of the microbial community on the incidence of clinical and subclinical mastitis, the following seven variables were defined and separately computed: a) the percentage of subclinical mastitis events on all records; b) the occurrence of subclinical mastitis at the moment of sample collecting (that is the CMT test result from 0 to 3); c) the number of mastitis treatments occurred during the lactation before dry-off; d) the number of clinical mastitis occurred in all lactations; e) the percentage of subclinical mastitis events with CMT score 1; f) the percentage of subclinical mastitis events with CMT score 3; g) the percentage of subclinical mastitis with CMT score 3.

The seven variables were separately analyzed via a single-trait mixed model analysis^50^. For each variable, two different approaches were used, aiming to describe as best as possible the influence of the microbial diversity on the target variables.

Firstly, each OTU was separately included as a fixed covariate in the model, together with a set of fixed factors defined in preliminary analyses and the animal ID as a random term. The fixed effects included the udder quarter, the age of the cow at recording, the day of recording, the duration (days) of the lactation. A series of 48 models, one for each OTU, was run for each y term. P-values were corrected for multiple comparisons using the Benjamini & Hochberg test^51^. Additionally, the other two models, considering either the Shannon index as a covariate instead of the single OTUs were run.

In a second step, the single OTUs were grouped in a number of factor scores to be used as a covariate. Factor scores are the output of a factorial analysis^52^ a technique for multivariate analyses able to remove redundant information from correlated traits (in this case, the OTUs) and synthesize the original traits in a smaller set of derived traits named ‘factors’, each including traits with common biological and/or physiological meaning ^53,54^. An exploratory factor analysis with Varimax rotation^55^ was performed on OTUs abundance to retain an amount of 18 factors according to the criterion of eigenvalues ≥1 (i.e., Kaiser criterion)^56^. Since factors are orthogonal and uncorrelated, they were all introduced in the same model as covariate without problems of collinearity. The model also included the same fixed factors and the animal ID of the previous analysis. This modeling allowed us to consider the variability of the OTUs abundance at one time.

For both steps, the statistical significance of the OTUs covariate or Shannon indexes (step 1) or of the factor scores included (step 2) was detected considering the result of the test for fixed effects performed in the mixed model analysis. A p-value ≤ 0.05 was defined for statistically significant results, and a p-value ≤ 0.10 was retained to detect a tendency to significance. The linear regression coefficient of each covariate on the target trait was used to study whether the target variable and OTU abundance varied in the same or in opposite directions.

## Results and Discussion

### Milk bacterial composition at dry-off

**Figure 1:**
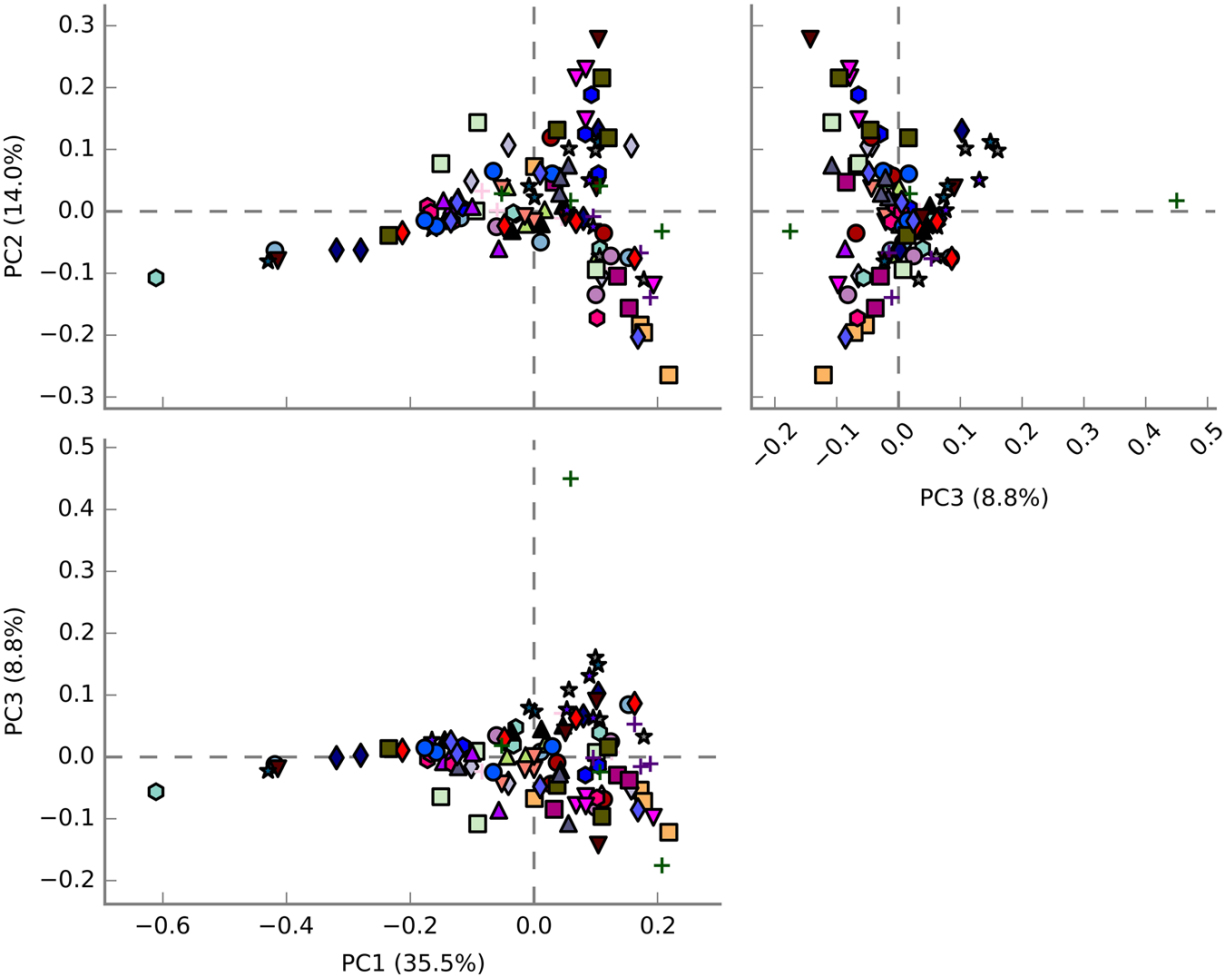
Plot of the three principal components (PC) determined after principal component analysis (PCA) of 16S rRNA data from microbiomes associated with milk samples collected at dry-off from October 2015 to July 2016. Mammary quarters from the same animal are represented by equal symbols with the same color.

To obtain a comprehensive characterization of the milk microbial composition at dry-off and to evaluate the presence of the genus *Staphylococcus*, we conducted a deep amplicon sequencing of the 16S rRNA gene (V4 region). Primers used for PCR amplification matched both bacterial and archaeal 16S rRNA gene, however, the archaeal composition was only taken into consideration to calculate principal components (PCA). PCA analysis did not evidence major differences among the 109 teats at dry-off. PC1, PC2 and PC3 explain more than 58% of the variability (Fig. 1).

**Figure 2:**
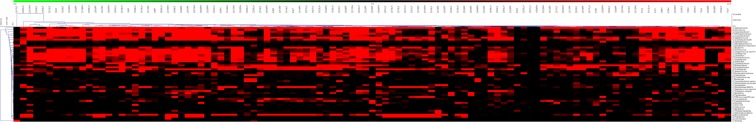
Hierarchical cluster analysis (HCA) and heat map of each animal quarter (n = 109) based on the relative abundance (> 0.5%) of the most abundant OTUs identified in milk samples of dry cows. Green to red gradient indicates low to high relative levels of OTUs within the given taxonomic unit.

After the quality control and clustering process at 3% divergence, 48 OTUs accounting for 95% relative abundance were assigned. The grouping of the quarters based on their most abundant OTUs revealed a dissimilar bacterial community among the teats (Fig. 2). This observation might be due to variations in the milk from different quarters of the same udder, which are separated by the presence of connective tissue.

Rarefaction curves tend to reach a plateau, suggesting that the sequencing depth was enough to cover the microbial diversity of samples. Indeed, approximately 63% of the samples displayed Good’s coverage index above 80%. The number of sequences, rarefaction curves and biodiversity indices associated with each sample is reported in Supplementary Spreadsheet S1.

At dry-off, seven phyla resulted to be the most abundant, namely *Firmicutes*, *Proteobacteria*, *Actinobacteria*, *Bacteroidetes*, *Crenarchaeota*, *Thermotogae* and *Cyanobacteria* (Supplementary Fig. S2). Our findings regarding the relative abundance of bacterial phyla are supported by previous studies^4,5^, which evaluated the dry cow therapy and the impact of weather in the raw milk microbiome of dairy cows. In total, the present study identified 27 major bacterial genera, among which *Sphingomonas*, *Corynebacterium*, *Staphylococcus* and *Aerococcus* constituted the prevalent ones. According to Quigley *et al*.^57^, these genera are commonly found in bovine and human raw milk.

We investigated the effect of the most abundant OTUs on the incidence of clinical and subclinical mastitis considering seven variables via single-trait mixed model analysis. In this study, the genus *Acinetobacter* is strongly and positively associated with the percentage of subclinical mastitis events scored as CMT 3, and in the total percentage of subclinical mastitis at the moment of sample collection (Supplementary Spreadsheet S2 – Tables S2b and S2g), but not in occurrence of clinical mastitis (Supplementary Spreadsheet S2 – Tables S2c and S2d). In the other conditions the presence of *Acinetobacter* was not significant after correction for multiple comparisons. Our results are in agreement with Patel *et al*.^58^, which reported the depletion of the genus *Acinetobacter* in human milk samples obtained from subacute and acute mastitis. Some genera resulted not significantly associated with some of the variables considered after multiple comparisons correction applied to the single-trait analysis, but they became significant when included in factor scores (further considerations on factor analysis are reported below). The genus *Staphylococcus* was highly correlated with the percentage of subclinical mastitis events on all records and at the moment of sampling, and on the CMT scored as 3 when included in Factor 11, together with *Acinetobacter* (Supplementary Spreadsheet S3 – Tables S3a and S3g). Although 16S rRNA analysis used in this study cannot reach the species level, our findings regarding the genus *Staphylococcus* are of major importance (third most abundant OTU) and *S. aureus* isolates were obtained from milk samples. As discussed below, a representative genome of *S. aureus* was selected and a comparative genomic investigation was performed with another strain isolated from acute clinical mastitis in order to identify genes associated with the colonization ability.

The genera *Sphingomonas* and *Exiguobacterium*, both included in Factor 5, also explained the percentage of subclinical mastitis events on all records and the CMT scores 2 and 3, as well as the number of mastitis treatments occurred during the lactation before dry-off (Supplementary Spreadsheet S3 – Tables S3a, S3c, S3f and S3g). The last genus encompasses atypical halophilic species found in goat milk^59^. *Sphingomonas* sp. is a Gram-negative, aerobic and non-spore forming psychrotrophic bacteria with high relative abundance in clinical mastitis quarters^60^. In fact, in our analysis *Sphingomonas* represents the most abundant genus taking into consideration cows that underwent antibiotic therapy to treat clinical mastitis during their life, being accompanied by the genus *Brevundimonas*, commonly found in milks from clinical mastitis^61^ (Supplementary Spreadsheet S2 – Table S2d; Supplementary Spreadsheet S3 – Tables S3c and S3d). It is worth mentioning that *Sphingomonas* spp. is implicated in the initiation of biofilm establishment on reverse osmosis membranes, frequently found in drinking water distributions systems, and isolated from gaskets, floors and walls of cheese-making factories^62,63^. As described elsewhere^64,65^, bacterial biofilm formation capability is considered a relevant factor involved in chronic udder infections and treatment failure. Our data suggest that *Sphingomonas* spp. is a component of the complex bacterial community involved in determining this disease, and it might be involved in biofilm development in dairy cows with subclinical mastitis.

Overall, microbial diversity at drying off did not affect the occurrence of subclinical or clinical mastitis, being the covariates for Shannon indexes not significant (data not shown).

**Figure 3:**
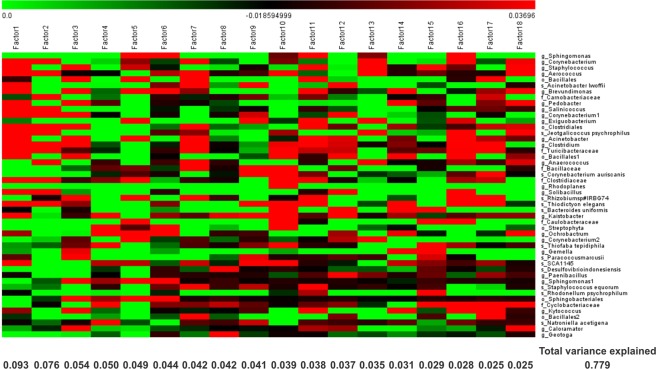
Factorial analysis considering the abundance of information of the most abundant taxa (> 0.5%). The variance explained by each factor is displayed on the bottom of the corresponding column. Green to red gradient indicates low to high magnitude of the variability obtained for each factor (loading).

Factor analysis allowed in one shot a detailed investigation of the abundant information obtained for all taxa, because the factors, each one grouping a number of OTUs, are orthogonally independent, as above mentioned. OTUs abundance at one time considering 18 factors explained approximately 78% of the variability and is displayed in Fig. 3.

In general, factor analysis showed that clinical mastitis events during lactation before dry-off were positively associated with the abundance of nine different taxa (Supplementary Spreadsheet S3). Among them, the presence of some uncommon microorganisms such as *Pedobacter*, *Rhizobium sp#IRBG74*, *Paracoccus marcusii* and *Ochrobactrum* are common in the dairy environment and can be isolated from soil and water, along with bulk milk tank^4,57,66,67^. The recent identification of numerous antimicrobial resistance mechanisms associated with *Pedobacter* species is giving rise to concern, since these genetic traits can potentially be horizontally transferred to other species^68^. Notwithstanding *Corynebacterium* spp. has been frequently isolated from subclinical mastitis, our study highlights a positive correlation between this genus and clinical mastitis records. Using a longitudinal transmission model, Rachah *et al*.^69^ demonstrated an increase of new IMI caused by previous udder infections determined by *Corynebacterium* spp., which might explain the outbreaks of clinical mastitis.

Lastly, the genus *Solibacillus* is a component of the core microbiota of water buffalo milk and it was previously found that its relative abundance decreased in milk from subclinical mastitis when compared with milk of healthy quarters^70^. This study reveals that *Solibacillus* is also present in dairy cows with healthy udders and its relative abundance is increased in the milk of animals identified with subclinical mastitis using CMT.

### Antimicrobial susceptibility by disc diffusion assay and MIC

The antibiotic sensitivity profile of *S. aureus* 3059 and UFV2030RH1 was evaluated by means of disc diffusion and minimum inhibitory concentration (MIC) assays. Considering a set of 25 different antibiotics, both strains showed high susceptibility to almost all antibiotics, except to aztreonam and bacitracin, which reflects a low level of antibiotic resistance in terms of *S. aureus*.

The resistance of *S. aureus* to aztreonam is not surprising, since this antibiotic is very effective on Gram-negative bacteria but not on Gram-positive and/or anaerobic species^71^, whereas bacitracin demonstrates activity mainly against Gram-positive bacteria^72^. Fifteen years ago, bacitracin resistance was uncommon and only a small number of cases have been reported, however this antimicrobial has become one of the most common drugs used in livestock as growth promoters in Brazil as well as a popular topical antibiotic. In fact, a Brazilian survey on dairy farms in the southern regions reported nearly 43% of bacitracin resistance in *Staphylococcus* spp. (n = 30) isolates^73^. A similar resistance rate (44%) was reported for *S. aureus* (n = 50) isolated from cattle and pigs slaughtered in South African slaughterhouses^74^.

Minimum inhibitory concentration (MIC) assay was performed with three antimicrobials commonly used for dry cow therapy, namely cephalonium, ceftiofur and ampicillin. For *S. aureus* 3059, values of 0.125 µg/mL, 1 µg/mL and 2 µg/mL were obtained, respectively, whereas values of 0.25 µg/mL, 2 µg/mL and 8 µg/mL were determined for the isolate *S. aureus* UFV2030RH1, correspondingly. The presence of multi-resistance traits in *Staphylococcus* spp. originating from dairy cows is routinely described in the literature and determined using different assays (disk diffusion, MIC and *in silico* analyses)^73,75–77^. By monitoring cephalonium susceptibility of udder pathogens throughout Europe, de Jong *et al*.^78^ reported MIC_50_ and MIC_90_ values of 0.12 and 0.25 µg/mL for *S. aureus* (n = 192). These values are close to the findings reported in the present study, and due to the absence of veterinary-specific breakpoints, it was not possible to discriminate between susceptibility and resistance. Regarding ceftiofur, MIC values of 1 and 2 µg/mL were identified for *S. aureus* 3059 and UFV2030RH1, respectively. Both values are lower than the so-called “resistant-breakpoint” (resistance ≥ 8 µg/mL) based on the Clinical and Laboratory Standards Institute (CLSI)^27^. According to MIC assays, values determined for *S. aureus* 3059 and UFV2030RH1 using ampicillin were the highest, and sixteen times higher than the MIC resistance breakpoint (≥0.5 µg/mL)^78^. High resistance to ampicillin among *S. aureus* strains isolated from dairy farms are frequently reported in the literature, surprisingly overcoming 80% of the total isolates in some studies; this finding reflects its widespread use and dispersion in the dairy environment^79,80^. Together, our results suggest that a low antibiotic resistance level was found in this herd, although special attention must be given to ampicillin. This is probably due to antibiotic management adopted during the last years on this farm which has taken into consideration the use of different antimicrobial classes based on individual clinical or subclinical mastitis history.

### **General features of*S. aureus*3059 and UFV2030RH1 genomes**

After quality filtering and merging of the overlapping PE reads, a total of 2,394,574 and 2,244,957 sequences, with an average length of 161 bp, were obtained and provided nearly 126 and 148-fold genome coverage for *S. aureus* 3059 and UFV2030RH1, respectively. The assembled sequences resulted in 30 and 38 scaffolds, with a total length of 2,709,051 and 2,735,181 bp and an average G + C content of 32.8 and 32.7% for *S. aureus* 3059 and UFV2030RH1, correspondingly.

For *S. aureus* 3059 and UFV2030RH1, the RAST server predicted 2,586 coding sequences (CDSs) for *S. aureus* 3059 and 2,607 for *S. aureus* UFV2030RH1, with an average of 37% of the genes assigned to subsystems (Supplementary Fig. S3). Functional categorization using GhostKOALA annotated 53.1% (*S. aureus* 3059) and 54.1% (*S. aureus* UFV2030RH1) of the total proteins in twenty-two functional groups (Supplementary Fig. S4). Overall, relevant differences were noticed in categories associated with the metabolism of carbohydrate, nucleotide, amino acids and cofactors, cell regulation/signaling and bacteriophages.

Regarding mobile elements, two prophage regions (one intact and one questionable) have been identified in *S. aureus* 3059, namely PT1-3059 and PT2-3059. These DNA portions had a remarkable similarity (95% and 98%) with Bacteriophage 92 (GenBank accession number AY954967.1) and *Staphylococcus* phage tp310-2 (GenBank accession number EF462198.1), respectively. With regard to *S. aureus* UFV2030RH1, four prophage regions were predicted (two intact and two incomplete). However, only one prophage sequence showed high similarity with bacteriophages deposited in the GenBank database. The siphovirus UFV2030RH1-PT1 displayed an identity of 100% with the virus *Staphylococcus* phage JS01 (GenBank accession number KC342645.2). Recent studies demonstrated that prophages are widespread in *S. aureus* genomes and can confer genome plasticity, as well as increase the number of virulence factors^17,81^.

**Figure 4:**
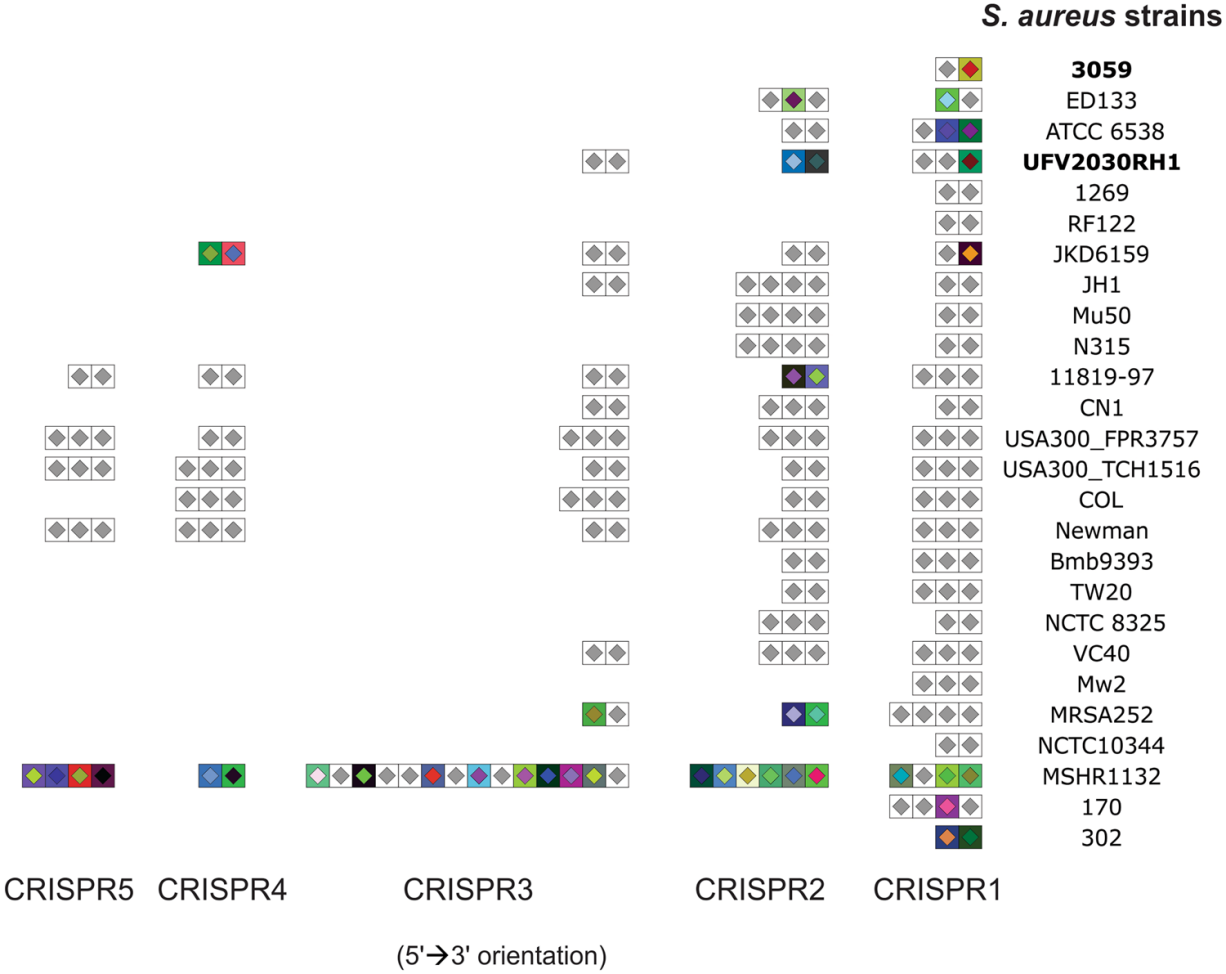
Prediction and comparison of CRISPR arrays present in 26 *S. aureus* strains. Similar spacers across and within the samples are colored in grey. No CRISPR locus was identified for *S. aureus* JH9, ST398 and 1364.

We also predicted and compared CRISPR arrays present in *S. aureus* UFV2030RH1 and 3059 with those found in 27 *S. aureus* reference genomes. The CRISPR and their *cas* (CRISPR-associated) genes include the so-called CRISPR-Cas system, an adaptive and heritable defense mechanism against phages and plasmids in bacteria and archaea. CRISPR typing either gives a phage resistance profile and is chronologically associated with bacteriophage infection^44^. Overall, the number of CRISPR loci identified among the 29 *S. aureus* strains involved in this analysis evidenced a remarkable heterogeneity, though a notable similarity of spacers across and within the strains has been identified (Fig. 4). For *S. aureus* UFV2030RH1, we identified three CRISPR arrays, whereas only one CRISPR cluster was identified in *S. aureus* 3059. Analysis of the unique spacers identified in UFV2030RH1, the CRISPR1_spacer 1, evidenced a high similarity score with nucleotide sequences acquired from the siphoviruses *Staphylococcus phage* YMC/09/04/R1988 (Genbank accession number NC_022758) and *Staphylococcus* phage phiNM3 (Genbank accession number NC_008617.1). Considering the CRISPR2, spacers 1 and 2 showed high similarity with nucleotide sequences found in *Megavirus lba* isolate LBA111 (Genbank accession number JX885207.1) and Vibriophage VpV262 (Genbank accession number AY095314), respectively. Despite only one spacer was identified in *S. aureus* 3059 (CRISPR1_spacer1), no matches were found on the PHAST database. Additionally, the presence of *cas* genes surrounding CRISPR loci has been investigated in order to select the intact CRISPR-cas systems. Only *S. aureus* MSHR1132 displayed *cas1* and *cas2* (Genbank accession numbers CCE57913, CCE57905 and CCE57906). A low number of *cas* genes (present in 3 out of 38 *S. aureus* strains) was also reported by Zhao *et al*.^82^ and it can be associated with failure in spacer acquisition. This is an interesting feature for phage therapy, since the functional CRISPR-Cas system is one of the most efficient phage defense pathways in bacteria.

The search for resistance determinants and associated antibiotics revealed major differences between *S. aureus* UFV2030RH1 and 3059. This finding was obtained considering only hits with weak similarity (named Loose) against antimicrobial resistance (AMR) genes, which can provide the detection of new genes (Supplementary Fig. S5). Among them, the antibiotic efflux category appears the most dissimilar in comparison with all the categories analyzed. Multidrug efflux pumps received special attention in the past years due to their potential association with clinical multidrug resistance^83^ Furthermore, recent advances demonstrate an important role of multidrug efflux pumps with biofilm formation, both mechanisms regulated by quorum-sensing mechanisms^84^. Since multidrug-resistant bacteria and biofilms have been the main targets in phage therapy^85,86^, these findings can reinforce the importance of phage study for more efficient control of *S. aureus* in mastitis.

### **Genomic comparison among*S. aureus*strains**

**Figure 5:**
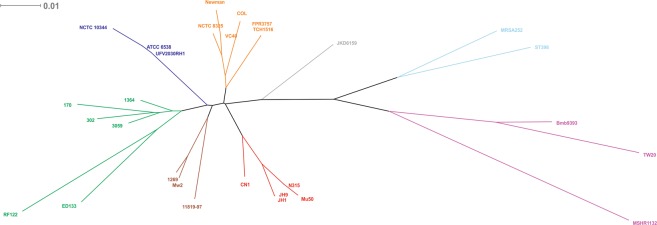
Phylogenomic tree constructed using the whole genome sequence of 29 *S. aureus* strains. GenBank accession numbers are reported in Supplementary Table S1. The scale bar represents a 1% difference in the average tBLASTx score. Clusters are identified by different colors.

From a whole-genome comparison, we constructed a phylogenetic tree to understand the genetic relationship between *S. aureus* 3059 and UFV2030RH1, also including reference strains obtained from different sources. Notwithstanding a 95% similarity between the two strains, they were grouped in different clusters (Fig. 5).

Interestingly, the genotype *S. aureus* 3059 clustered with *S. aureus* 302 and 170, both isolated from dairy cows with persistent and transient subclinical mastitis, respectively^87^. Although several genotypes of *S. aureus* have been identified and associated with high-herd prevalence^88,89^, most isolates can share up to 78% of conserved genes (core genome) independently from their isolation source^90^. Nowadays, it is reasonable to infer that host susceptibility, the presence of core-variable genes (e.g *sdrD*, *clfA-B*, *sasD* and *fnbB*) and their expression level are associated with the severity of mastitis^91,92^.

*S. aureus* UFV2030RH1 displayed high identity with the methicillin-sensitive *S. aureus* NCTC 10344 and ATCC 6538, both isolated from humans. The latter strain has been commonly used as a standard strain to test disinfectants and other antimicrobial compounds^93^. Strain ATCC 6538 also possesses a plasmid of 28,078 bp, which is not present in UFV2030RH1. This finding suggests that the herd involved in this study may have acquired UFV2030RH1 by a human-to-cow transferring mechanism.

The use of comparative tools using RAST allowed us to confront the functional parts of *S. aureus* 3059 and UFV2030RH1. This analysis considered the set of proteins that are unique to each strain (Table 1). Apart from sequences specific for prophages (discussed above), genes coding for microbial surface components recognizing adhesive matrix molecules (MSCRAMM) proteins were found in the UFV2030RH1 genome and this is one of the main differences between the two isolates. Considered as members of the “core variable” genes, *sdrD* and *sasG* encode proteins that promote adhesion/colonization to host cells and contribute to biofilm formation^91,94^. Indeed, *S. aureus* UFV2030RH1 has demonstrated a stronger capability to adhere and form biofilms in bovine mammary epithelial cells (MAC-T) when compared to *S. aureus* 3059 (unpublished data).

**Table 1:** Functioning comparison between *S. aureus* 3059 and UFV2030RH1.

Strain	Category	Subcategory	Subsystem	Role	KEGG
UFV2030RH1	DNA Metabolism	*	Restriction-Modification System	Type I restriction-modification system, specificity subunit S	*
	Phages, Prophages, Transposable elements, Plasmids	Phages, Prophages	Phage capsid proteins	Phage capsid protein	*
			Phage packaging machinery	Phage DNA packaging	*
				Phage DNA-binding protein	*
			Phage replication	Phage replication initiation protein	*
	Virulence, Disease and Defense	Adhesion	Adhesins in *Staphylococcus*	Adhesin of unknown specificity (*sdrD*)	K14194
				Virulence-associated cell-wall-anchored protein SasG (LPXTG motif)	K14195
	Carbohydrates	Central carbohydrate metabolism	Pyruvate metabolism I: anaplerotic reactions, PEP	Malolactic enzyme	K00382
3059	DNA Metabolism	*	CBSS-1352.1.peg.856	Transcriptional regulator, PadR family	K02986
		DNA repair	DNA repair, bacterial	DNA-cytosine methyltransferase	K19080
	Phages, Prophages, Transposable elements, Plasmids	Phages, Prophages	Phage capsid proteins	Phage capsid and scaffold	*
				Phage major capsid protein	*
				Phage minor capsid protein	*
	Regulation and Cell signaling	*	cAMP signaling in bacteria	Prophage Clp protease-like protein	*
	Virulence, Disease and Defense	Resistance to antibiotics and toxic compounds	Fosfomycin resistance	Fosfomycin resistance protein FosB	*

Only the set of proteins that are unique to each strain is shown. The asterisk indicates a lack of information according to the RAST and KEGG databases.

### vB_SauM-UFV_DC4

The virus UFV_DC4 was isolated from dairy cattle farm waste collected from the same environment in which the dairy cows enrolled in this study were stabled. This procedure allowed the isolation of a new bacteriophage having as potential targets the *S. aureus* strains present in this dairy herd.

**Figure 6:**
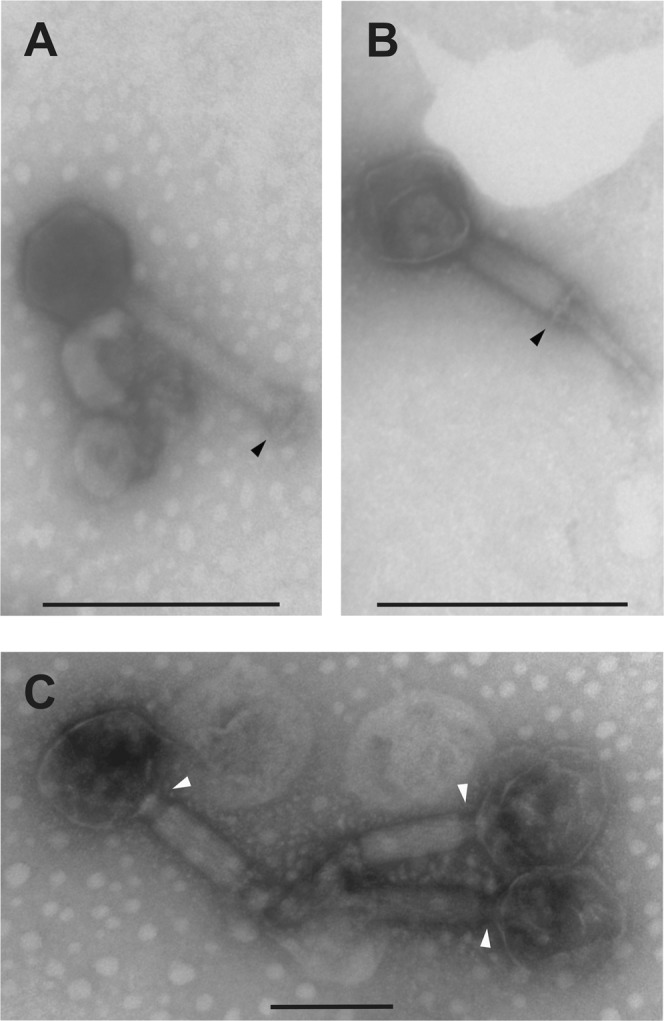
Transmission electron microscopy of vB_SauM-UFV_DC4. The black head arrow in (**A**) indicates the tail in native conformation, whereas we observe a double-layered baseplate in (**B**) after tail contraction. In (**C**), white head arrows show structural changes of the neck connector that occurred after tail contraction.

Electron microscopy of UFV_DC4 revealed a non-icosahedral head (width 90 nm; length 102 nm) and a long, contractile, double-sheathed tail of 180 nm, belonging to the family *Myoviridae*, order *Caudovirales* (Fig. 6). In this study, we adopted the nomenclature proposed by Kropinski *et al*.^95^, and in our code “v” is the abbreviation for “virus”, “B” for “bacteria”, “Sau for “*Staphylococcus aureus*”, “M” for “Myoviridae” and “UFV_DC4” is our own code. This phage has an unusual longer neck with respect to the other phages of the *Myoviridae* family (Fig. 6C).

## Conclusion

Bovine mastitis caused by *S. aureus* still can cause significant losses for dairy farmers. This study revealed that *Staphylococcus* was the third most present bacterial genus considering 109 teats of healthy Holstein cows chose at the dry-off period. Furthermore, *Staphylococcus* spp. was highly correlated with subclinical mastitis events presenting CMT scored as 2 and 3. The genome sequencing of *S. aureus* UFV2030RH1 showed the presence of protein-coding genes associated with adhesive matrix molecules that recognize microbial surface components, which can be implicated in chronic mastitis. The capability of vB_SauM-UFV_DC4 to lyse *S. aureus* 3059 and UFV2030RH1 cells, respectively involved in clinical and subclinical mastitis, can bring further insights into the comprehension of lytic phages for *S. aureus* with potential use in phage therapy.

## Supplementary information

Supplementary figures S1-S5.

Supplementary tables S1-S2.

Supplementary Spreadsheet S1.

Supplementary Spreadsheet S2.

Supplementary Spreadsheet S3.

## References

[1] OECD/FAO. *OECD-FAO Agricultural Outlook 2016-2025*. *Oecd/Fao*, 10.1787/agr_outlook-2016-en (OECD, 2016).

[2] Guimarães JLB (2017). Estimate of the economic impact of mastitis: A case study in a Holstein dairy herd under tropical conditions. Prev. Vet. Med..

[3] Scherpenzeel CGM, Hogeveen H, Maas L, Lam TJGM (2018). Economic optimization of selective dry cow treatment. J. Dairy Sci..

[4] Bonsaglia ECR (2017). Milk microbiome and bacterial load following dry cow therapy without antibiotics in dairy cows with healthy mammary gland. Sci. Rep..

[5] Li N (2018). Variation in Raw Milk Microbiota Throughout 12 Months and the Impact of Weather Conditions. Sci. Rep..

[6] Taponen S (2019). Bovine milk microbiome: A more complex issue than expected. Vet. Res..

[7] Hoque MN (2019). Metagenomic deep sequencing reveals association of microbiome signature with functional biases in bovine mastitis. Sci. Rep..

[8] Shaheen M, Tantary H, Nabi S (2016). A Treatise on Bovine Mastitis: Disease and Disease Economics, Etiological Basis, Risk Factors, Impact on Human Health, Therapeutic Management, Prevention and Control Strategy. Adv. Dairy Res..

[9] Crispie F, Flynn J, Ross RP, Hill C, Meaney WJ (2004). Dry cow therapy with a non-antibiotic intramammary teat seal - a review. Ir. Vet. J..

[10] Vanhoudt, A. *et al*. Effects of reduced intramammary antimicrobial use during the dry period on udder health in Dutch dairy herds. *J. Dairy Sci*., 10.3168/jds.2017-13555 (2018). 10.3168/jds.2017-13555 29395142

[11] Rajala-Schultz PJ, Torres AH, DeGraves FJ (2011). Milk yield and somatic cell count during the following lactation after selective treatment of cows at dry-off. J. Dairy Res..

[12] da Silva Duarte V (2018). Genomic analysis and immune response in a murine mastitis model of vB_EcoM-UFV13, a potential biocontrol agent for use in dairy cows. Sci. Rep..

[13] Gill JJ (2006). Efficacy and Pharmacokinetics of Bacteriophage Therapy in Treatment of Subclinical *Staphylococcus aureus* Mastitis in Lactating Dairy Cattle. Antimicrob. Agents Chemother..

[14] da Silva Duarte, V. *et al*. A T4virus prevents biofilm formation by *Trueperella pyogenes*. *Vet. Microbiol*. **218** (2018). 10.1016/j.vetmic.2018.03.025 29685220

[15] Dias RS (2013). Use of phages against antibiotic-resistant *Staphylococcus aureus* isolated from bovine mastitis1. J. Anim. Sci..

[16] Rodríguez-Rubio, L., Martínez, B., Donovan, D. M., García, P. & Rodríguez, A. Potential of the Virion-Associated Peptidoglycan Hydrolase HydH5 and Its Derivative Fusion Proteins in Milk Biopreservation. *PLoS One* **8** (2013). 10.1371/journal.pone.0054828 PMC3554637 23359813

[17] Deghorain M, Van Melderen L (2012). The *Staphylococci* Phages Family: An Overview. Viruses.

[18] Cui, Z. *et al*. Safety assessment of *Staphylococcus* phages of the family *Myoviridae* based on complete genome sequences. *Sci. Rep*., 10.1038/srep41259 (2017). 10.1038/srep41259 PMC5259776 28117392

[19] Abatángelo V (2017). Broad-range lytic bacteriophages that kill *Staphylococcus aureus* local field strains. PLoS One.

[20] Verstappen, K. M. *et al*. The effectiveness of bacteriophages against methicillin-resistant *Staphylococcus aureus* ST398 nasal colonization in pigs. *PLoS One*, 10.1371/journal.pone.0160242 (2016). 10.1371/journal.pone.0160242 PMC4972443 27487020

[21] Hsu, B. B. *et al*. Dynamic Modulation of the Gut Microbiota and Metabolome by Bacteriophages in a Mouse Model. *Cell Host Microbe*, 10.1016/j.chom.2019.05.001 (2019). 10.1016/j.chom.2019.05.001 PMC6579560 31175044

[22] Zheng, D.-W. *et al*. Phage-guided modulation of the gut microbiota of mouse models of colorectal cancer augments their responses to chemotherapy. *Nat. Biomed. Eng*., 10.1038/s41551-019-0423-2 (2019). 10.1038/s41551-019-0423-2 31332342

[23] Synnott, A. J. *et al*. Isolation from sewage influent and characterization of novel *Staphylococcus aureus* bacteriophages with wide host ranges and potent lytic capabilities. *Appl. Environ. Microbiol*., 10.1128/AEM.02641-08 (2009). 10.1128/AEM.02641-08 PMC2704828 19411410

[24] Bourdin G (2014). Amplification and Purification of T4-Like *Escherichia coli* Phages for Phage Therapy: from Laboratory to Pilot Scale. Appl. Environ. Microbiol..

[25] Schneider, C. A., Rasband, W. S. & Eliceiri, K. W. NIH Image to ImageJ: 25 years of image analysis. *Nat. Methods* (2012). 10.1038/nmeth.2089 PMC5554542 22930834

[26] Lefkowitz, E. J. *et al*. Virus taxonomy: The database of the International Committee on Taxonomy of Viruses (ICTV). *Nucleic Acids Res*., 10.1093/nar/gkx932 (2018). 10.1093/nar/gkx932 PMC5753373 29040670

[27] CLSI. Performance Standards for Antimicrobial Disk and Dilution Susceptibility Tests for Bacteria Isolated from Animals, 3rd edition. *Clin. Lab. Stand. Inst*. (2015).

[28] Wiegand I, Hilpert K, Hancock REW (2008). Agar and broth dilution methods to determine the minimal inhibitory concentration (MIC) of antimicrobial substances. Nat. Protoc..

[29] Stevenson, D. M. & Weimer, P. J. Dominance of *Prevotella* and low abundance of classical ruminal bacterial species in the bovine rumen revealed by relative quantification real-time PCR. *Appl. Microbiol. Biotechnol*., 10.1007/s00253-006-0802-y (2007). 10.1007/s00253-006-0802-y 17235560

[30] Campanaro S, Treu L, Kougias PG, Zhu X, Angelidaki I (2018). Taxonomy of anaerobic digestion microbiome reveals biases associated with the applied high throughput sequencing strategies. Sci. Rep..

[31] Pospiech, A. & Neumann, B. A versatile quick-prep of genomic DNA from Gram-positive bacteria. *Trends Genet*., 10.1016/S0168-9525(00)89052-6 (1995). 10.1016/s0168-9525(00)89052-6 7638902

[32] Caporaso JG (2010). QIIME allows analysis of high-throughput community sequencing data. Nat. Methods.

[33] Bolger AM, Lohse M, Usadel B (2014). Trimmomatic: a flexible trimmer for Illumina sequence data. Bioinformatics.

[34] Edgar RC (2010). Search and clustering orders of magnitude faster than BLAST. Bioinformatics.

[35] Langmead B, Salzberg SL (2012). Fast gapped-read alignment with Bowtie 2. Nat. Methods.

[36] Li H (2009). The Sequence Alignment/Map (SAM) Format and SAMtools 1000 Genome Project Data Processing Subgroup. Bioinformatics.

[37] Howe, E. *et al*. MeV: MultiExperiment Viewer. in *Biomedical Informatics for Cancer Research* (eds. Ochs, M. F., Casagrande, J. T. & Davuluri, R. V) 267–277, 10.1007/978-1-4419-5714-6_15 (Springer US, 2010).

[38] Parks DH, Tyson GW, Hugenholtz P, Beiko RG (2014). STAMP: Statistical analysis of taxonomic and functional profiles. Bioinformatics.

[39] Bosi, E. *et al*. MeDuSa: A multi-draft based scaffolder. *Bioinformatics*, 10.1093/bioinformatics/btv171 (2015). 10.1093/bioinformatics/btv171 25810435

[40] Aziz, R. K. *et al*. The RAST Server: Rapid annotations using subsystems technology. *BMC Genomics*, 10.1186/1471-2164-9-75 (2008). 10.1186/1471-2164-9-75 PMC2265698 18261238

[41] Kanehisa M, Sato Y, Morishima K (2016). BlastKOALA and GhostKOALA: KEGG Tools for Functional Characterization of Genome and Metagenome Sequences. J. Mol. Biol..

[42] Arndt D (2016). PHASTER: a better, faster version of the PHAST phage search tool. Nucleic Acids Res..

[43] Biswas, A., Staals, R. H. J., Morales, S. E., Fineran, P. C. & Brown, C. M. CRISPRDetect: A flexible algorithm to define CRISPR arrays. *BMC Genomics*, 10.1186/s12864-016-2627-0 (2016). 10.1186/s12864-016-2627-0 PMC4869251 27184979

[44] Dion M, Labrie S, Shah S, Moineau S (2018). CRISPRStudio: A User-Friendly Software for Rapid CRISPR Array Visualization. Viruses.

[45] Zhou Y, Liang Y, Lynch KH, Dennis JJ, Wishart DS (2011). HAST: A Fast Phage Search Tool..

[46] Jia B (2017). CARD 2017: expansion and model-centric curation of the comprehensive antibiotic resistance database. Nucleic Acids Res..

[47] Ågren J, Sundström A, Håfström T, Segerman B (2012). Gegenees: Fragmented Alignment of Multiple Genomes for Determining Phylogenomic Distances and Genetic Signatures Unique for Specified Target Groups. PLoS One.

[48] Kloepper, T. H. & Huson, D. H. Drawing explicit phylogenetic networks and their integration into SplitsTree. *BMC Evol. Biol*., 10.1186/1471-2148-8-22 (2008). 10.1186/1471-2148-8-22 PMC2253509 18218099

[49] Hammer, Ø., Harper, D. A. T. a. T. & Ryan, P. D. PAST: Paleontological Statistics Software Package for Education and Data Analysis. *Palaeontol. Electron*., 10.1016/j.bcp.2008.05.025 (2001).

[50] SAS Institute Inc. Base SAS® 9.4 Procedures Guide. *Stat. Proced*. **Second Edi**, Cary, NC: SAS Institute Inc. (2013).

[51] Benjamini, Y. & Hochberg, Y. Controlling the False Discovery Rate: A Practical and Powerful Approach to Multiple Testing. *J. R. Stat. Soc. Ser. B*, 10.1111/j.2517-6161.1995.tb02031.x (1995).

[52] Thompson, B. Exploratory and confirmatory factor analysis: Understanding concepts and applications. Exploratory and confirmatory factor analysis: Understanding concepts and applications., 10.1037/10694-000 (American Psychological Association, 2004).

[53] Vukasinovic N, Moll J, Künzi N (1997). Factor analysis for evaluating relationships between herd life and type traits in Swiss Brown cattle. Livest. Prod. Sci..

[54] Ali AK, Koots KR, Burnside EB (1998). Factor Analysis of Genetic Evaluations for Type Traits of Canadian Holstein Sires and Cows. Asian-Australasian J. Anim. Sci..

[55] SAS Institute Inc. The MIXED Procedure. *SAS/STAT 9.2 User’s Guid*., 10.1136/bjsports-2015-095677 (2015).

[56] Russell DW (2002). In search of underlying dimensions: The use (and abuse) of factor analysis in. Personality and Social Psychology Bulletin. Personal. Soc. Psychol. Bull..

[57] Quigley L (2013). The complex microbiota of raw milk. FEMS Microbiol. Rev..

[58] Patel SH (2017). Culture independent assessment of human milk microbial community in lactational mastitis. Sci. Rep..

[59] Callon C (2007). Stability of microbial communities in goat milk during a lactation year: Molecular approaches. Syst. Appl. Microbiol..

[60] Oikonomou G (2014). Microbiota of Cow’s Milk; Distinguishing Healthy, Sub-Clinically and Clinically Diseased Quarters. PLoS One.

[61] Patel SH, Vaidya YH, Joshi CG, Kunjadia AP (2016). Culture-dependent assessment of bacterial diversity from human milk with lactational mastitis. Comp. Clin. Path..

[62] Bereschenko LA, Stams AJM, Euverink GJW, van Loosdrecht MCM (2010). Biofilm Formation on Reverse Osmosis Membranes Is Initiated and Dominated by *Sphingomonas* spp. Appl. Environ. Microbiol..

[63] Carpentier B, Chassaing D (2004). Interactions in biofilms between *Listeria monocytogenes* and resident microorganisms from food industry premises. Int. J. Food Microbiol..

[64] Schönborn S, Wente N, Paduch J-H, Krömker V (2017). *In vitro* ability of mastitis causing pathogens to form biofilms. J. Dairy Res..

[65] Melchior MB, Vaarkamp H, Fink-Gremmels J (2006). Biofilms: A role in recurrent mastitis infections?. Vet. J..

[66] Andrews M, Andrews ME (2017). Specificity in Legume-Rhizobia Symbioses. Int. J. Mol. Sci..

[67] Jayarao BM, Wang L (1999). A Study on the Prevalence of Gram-Negative Bacteria in Bulk Tank Milk. J. Dairy Sci..

[68] Viana AT, Caetano T, Covas C, Santos T, Mendo S (2018). Environmental superbugs: The case study of *Pedobacter* spp. Environ. Pollut..

[69] Dalen G (2018). Transmission dynamics of intramammary infections caused by *Corynebacterium* species. J. Dairy Sci..

[70] Catozzi C (2017). The microbiota of water buffalo milk during mastitis. PLoS One.

[71] Ramsey C, MacGowan AP (2016). A review of the pharmacokinetics and pharmacodynamics of aztreonam. J. Antimicrob. Chemother..

[72] Dowling, P. M. Peptide Antibiotics. in *Antimicrobial Therapy in Veterinary Medicine* 189–198, 10.1002/9781118675014.ch11 (John Wiley & Sons, Inc, 2013).

[73] Freitas, C. H. *et al*. Identification and antimicrobial suceptibility profile of bacteria causing bovine mastitis from dairy farms in Pelotas, Rio Grande do Sul. *Brazilian J. Biol*., 10.1016/j.physbeh.2005.08.044 (2018). 10.1590/1519-6984.170727 29319754

[74] Tanih, N. F., Sekwadi, E., Ndip, R. N. & Bessong, P. O. Detection of pathogenic *Escherichia coli* and *Staphylococcus aureus* from cattle and pigs slaughtered in abattoirs in Vhembe District, South Africa. *Sci. World J*., 10.1155/2015/195972 (2015). 10.1155/2015/195972 PMC4354961 25811040

[75] Yoshimura H, Ishimaru M, Kojima A (2002). Minimum Inhibitory Concentrations of 20 Antimicrobial Agents against *Staphylococcus aureus* Isolated from Bovine Intramammary Infections in Japan. J. Vet. Med. Ser. B.

[76] Oliveira L, Langoni H, Hulland C, Ruegg PL (2012). Minimum inhibitory concentrations of *Staphylococcus aureus* recovered from clinical and subclinical cases of bovine mastitis. J. Dairy Sci..

[77] Ster, C. *et al*. *In vitro* antibiotic susceptibility and biofilm production of *Staphylococcus aureus* isolates recovered from bovine intramammary infections that persisted or not following extended therapies with cephapirin, pirlimycin or ceftiofur. *Vet. Res*., 10.1186/s13567-017-0463-0 (2017). 10.1186/s13567-017-0463-0 PMC5609010 28934980

[78] Papich MG (2013). Antimicrobials, susceptibility testing, and minimum inhibitory concentrations (MIC) in veterinary infection treatment. Vet. Clin. North Am. Small Anim. Pract..

[79] Mekonnen, S. A. *et al*. Characterization of *Staphylococcus aureus* isolated from milk samples of dairy cows in small holder farms of North-Western Ethiopia. *BMC Vet. Res*., 10.1186/s12917-018-1558-1 (2018). 10.1186/s12917-018-1558-1 PMC6107951 30139356

[80] Watanabe, N., Bergamaschi, B. A., Loftin, K. A., Meyer, M. T. & Harter, T. Use and environmental occurrence of antibiotics in freestall dairy farms with manured forage fields. *Environ. Sci. Technol*., 10.1021/es100834s (2010). 10.1021/es100834s PMC2931405 20698525

[81] Diene, S. M. *et al*. Prophages and adaptation of *Staphylococcus aureus* ST398 to the human clinic. *BMC Genomics*, 10.1186/s12864-017-3516-x (2017). 10.1186/s12864-017-3516-x PMC5294865 28166723

[82] Zhao X, Yu Z, Xu Z (2018). Study the Features of 57 Confirmed CRISPR Loci in 38 Strains of *Staphylococcus aureus*. Front. Microbiol..

[83] Jang S (2016). Multidrug efflux pumps in *Staphylococcus aureus* and their clinical implications. J. Microbiol..

[84] Subhadra, B., Kim, D. H., Woo, K., Surendran, S. & Choi, C. H. Control of biofilm formation in healthcare: Recent advances exploiting quorum-sensing interference strategies and multidrug efflux pump inhibitors. *Materials*, 10.3390/ma11091676 (2018). 10.3390/ma11091676 PMC6163278 30201944

[85] Suresh MK, Biswas R, Biswas L (2019). An update on recent developments in the prevention and treatment of *Staphylococcus aureus* biofilms. Int. J. Med. Microbiol..

[86] Tkhilaishvili T, Lombardi L, Klatt A-B, Trampuz A, Di Luca M (2018). Bacteriophage Sb-1 enhances antibiotic activity against biofilm, degrades exopolysaccharide matrix and targets persisters of *Staphylococcus aureus*. Int. J. Antimicrob. Agents.

[87] Silva DM (2016). Draft Genome Sequences of *Staphylococcus aureus* Strains Isolated from Subclinical Bovine Mastitis in Brazil. Genome Announc..

[88] Cosandey, A. *et al*. *Staphylococcus aureus* genotype B and other genotypes isolated from cow milk in European countries. *J. Dairy Sci*., 10.3168/jds.2015-9587 (2016). 10.3168/jds.2015-9587 26585469

[89] Thiran E (2018). Biofilm formation of *Staphylococcus aureus* dairy isolates representing different genotypes. J. Dairy Sci..

[90] Feng Y (2008). Evolution and pathogenesis of *Staphylococcus aureus*: lessons learned from genotyping and comparative genomics. FEMS Microbiol. Rev..

[91] Capra, E. *et al*. Genomic and transcriptomic comparison between *Staphylococcus aureus* strains associated with high and low within herd prevalence of intra-mammary infection. *BMC Microbiol*., 10.1186/s12866-017-0931-8 (2017). 10.1186/s12866-017-0931-8 PMC5247818 28103794

[92] Shukla SK, Rose W, Schrodi SJ (2015). Complex host genetic susceptibility to *Staphylococcus aureus* infections. Trends Microbiol..

[93] Makarova, O., Johnston, P., Walther, B., Rolff, J. & Roesler, U. Complete Genome Sequence of the Disinfectant Susceptibility Testing Reference Strain *Staphylococcus aureus* subsp. *aureus* ATCC 6538. *Genome Announc*. **5** (2017). 10.1128/genomeA.00293-17 PMC5427206 28495771

[94] Liu J (2018). Transcriptomics Study on *Staphylococcus aureus* Biofilm Under Low Concentration of Ampicillin. Front. Microbiol..

[95] Kropinski, A. M., Prangishvili, D. & Lavigne, R. Position paper: The creation of a rational scheme for the nomenclature of viruses of Bacteria and Archaea. *Environ. Microbiol*., 10.1111/j.1462-2920.2009.01970.x (2009). 10.1111/j.1462-2920.2009.01970.x 19519870

