# Supplementary material for: Milk microbial composition of Brazilian dairy cows entering the dry period and genomic comparison between Staphylococcus aureus strains susceptible to the bacteriophage vB_SauM-UFV_DC4
Source: Sci Rep. 2020 Mar 26;10:5520. doi: 10.1038/s41598-020-62499-6 (PMC7099093; doi:10.1038/s41598-020-62499-6)

Milk microbial composition of Brazilian dairy cows entering the dry period and genomic comparison between *Staphylococcus aureus* strains susceptible to the bacteriophage vB_SauM-UFV_DC4

Vinícius da Silva Duarte^a, c^, Laura Treu^b,c^, Cristina Sartori^c^, Roberto Sousa Dias^a^, Isabela da Silva Paes^d^, Marcella Silva Vieira^d^, Gabriele Rocha Santana^d^, Marcos Inácio Marcondes^e^, Alessio Giacomini^c^, Viviana Corich^c^, Stefano Campanaro^b, f *^, Cynthia Silva^a^, Sérgio Oliveira de Paula^e^.

^a^ Department of Microbiology, Federal University of Viçosa, Av. Peter Henry Rolfs, s/n, Campus Universitário, 36570-900, Viçosa, Minas Gerais, Brazil

^b^ Department of Biology, University of Padova, Via U. Bassi 58/b, 35121, Padova, Italy

^c^ Department of Agronomy Food Natural Resources Animals and Environment, University of Padova, Viale dell’Universitá, 16, 35020 Legnaro (PD), Italy

^d^ Department of General Biology, Federal University of Viçosa, Av. Peter Henry Rolfs, s/n, Campus Universitário, 36570-900, Viçosa, Minas Gerais, Brazil

^e^ Department of Animal Science, Universidade Federal de Viçosa, Viçosa, Brazil

^f^ CRIBI Biotechnology Center Viale G. Colombo 3, 35121 Padova, Italy

*Address correspondence to Stefano Campanaro, stefano.campanaro@unipd.it


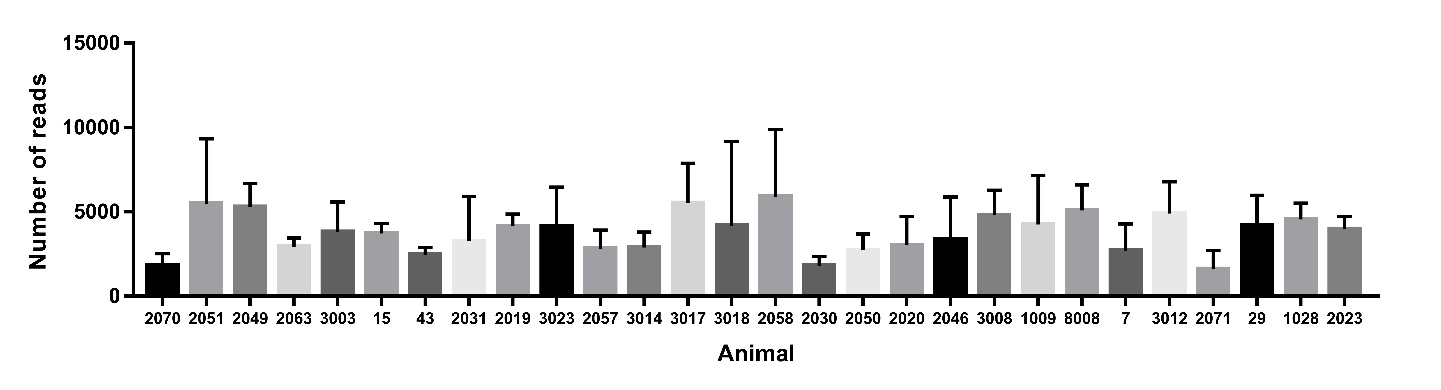


Supplementary Figure S1 – Average number of reads considering for each dairy cow.

Supplementary Figure S2. Relative abundance of the most abundant phyla at dry-off.


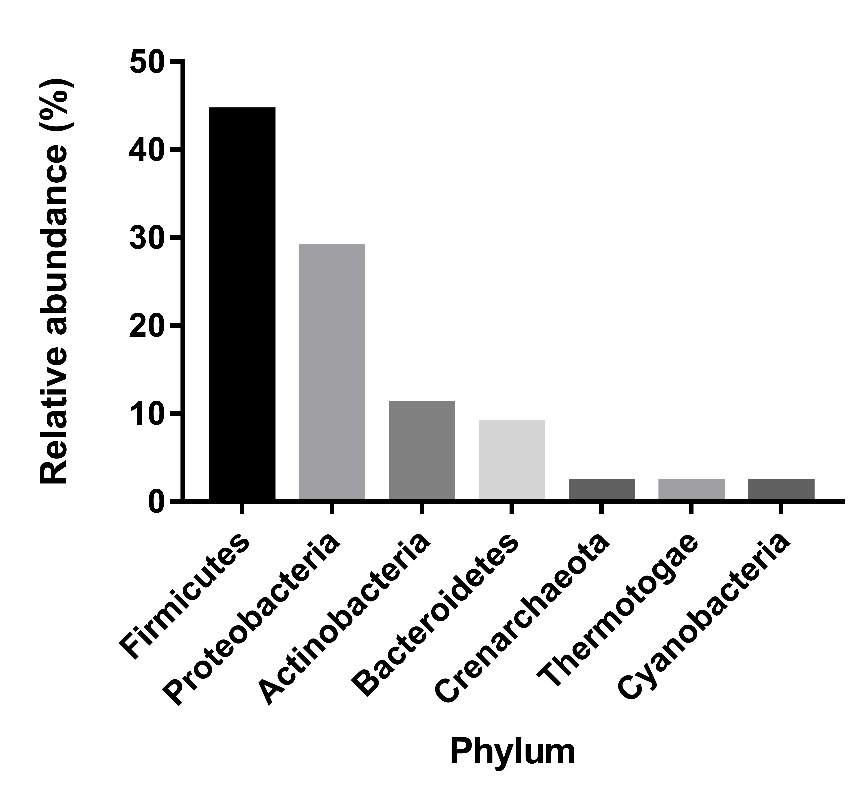


Supplementary Figure S3. RAST subsystem information for S. aureus 3059 (A) and S. aureus 2030RH1 (B).


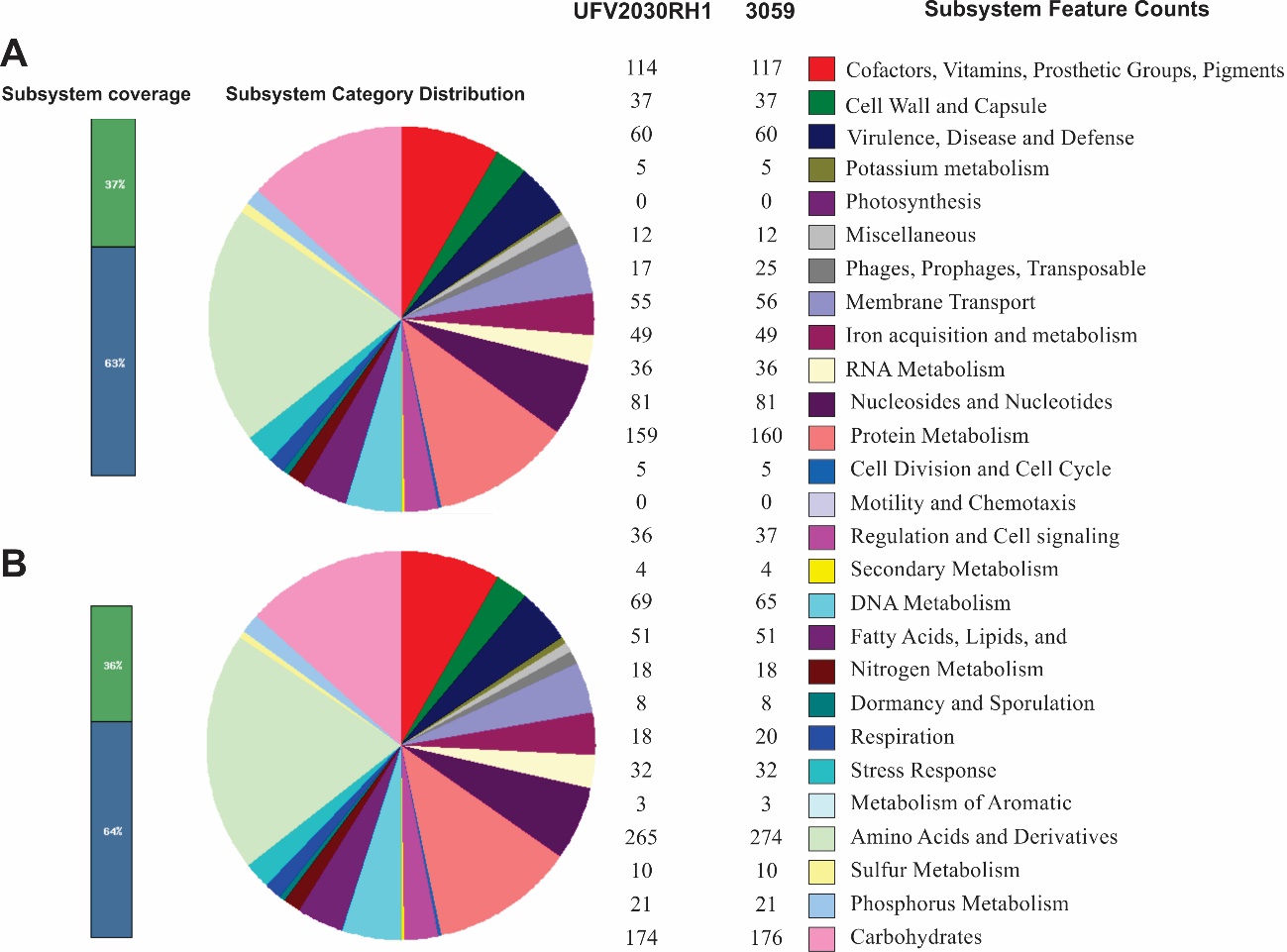


Supplementary Figure S4. Functional categorization using GhostKOALA for S. aureus 3059 (A) and S. aureus UFV2030RH1 (B).


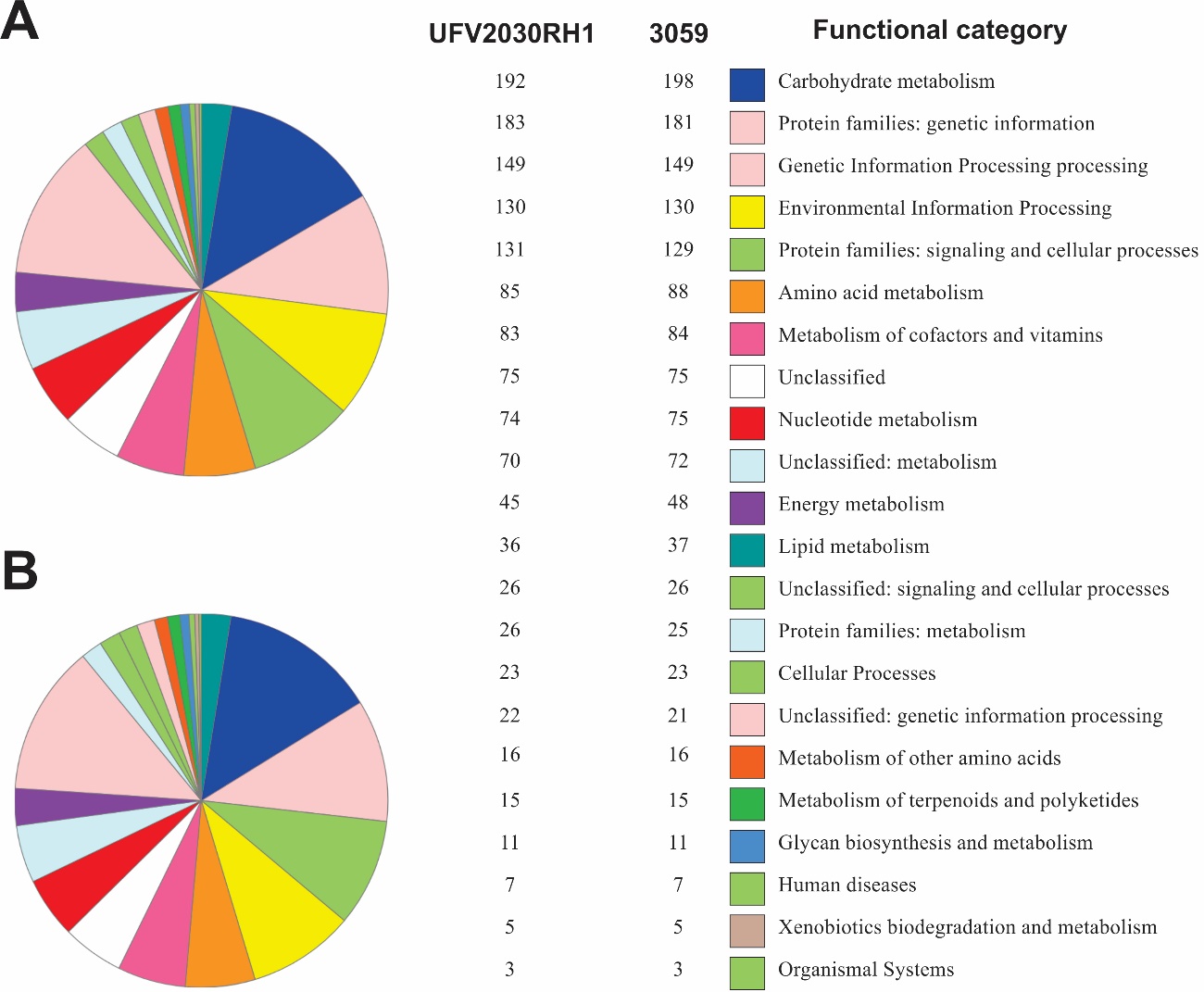


Supplementary Figure S5. In silico detection of antibiotic resistance genes in S. aureus 3059 (A) and S. aureus 2030RH1 using the Comprehensive Antibiotic Resistance Database (CARD)


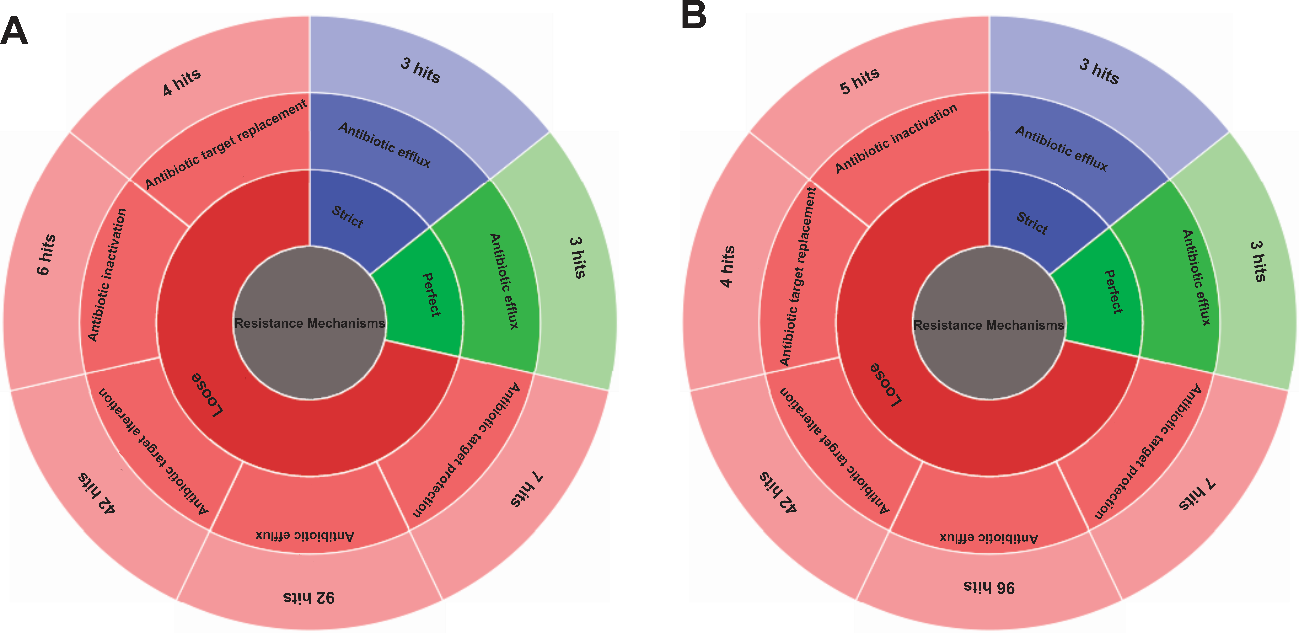

Supplement: Supplementary file 1 — Supplementary figures S1-S5. [file 41598_2020_62499_MOESM1_ESM.docx]
