# Supplementary material for: Milk microbial composition of Brazilian dairy cows entering the dry period and genomic comparison between Staphylococcus aureus strains susceptible to the bacteriophage vB_SauM-UFV_DC4
Source: Sci Rep. 2020 Mar 26;10:5520. doi: 10.1038/s41598-020-62499-6 (PMC7099093; doi:10.1038/s41598-020-62499-6)
Supplement: Supplementary file 2 — Supplementary tables S1-S2. [file 41598_2020_62499_MOESM2_ESM.docx]

Milk microbial composition of Brazilian dairy cows entering the dry period and genomic comparison between *Staphylococcus aureus* strains susceptible to the bacteriophage vB_SauM-UFV_DC4

Vinícius da Silva Duarte^a, c^, Laura Treu^b,c^, Cristina Sartori^c^, Roberto Sousa Dias^a^, Isabela da Silva Paes^d^, Marcella Silva Vieira^d^, Gabriele Rocha Santana^d^, Marcos Inácio Marcondes^e^, Alessio Giacomini^c^, Viviana Corich^c^, Stefano Campanaro^b, f *^, Cynthia Silva^a^, Sérgio Oliveira de Paula^e^.

^a^ Department of Microbiology, Federal University of Viçosa, Av. Peter Henry Rolfs, s/n, Campus Universitário, 36570-900, Viçosa, Minas Gerais, Brazil

^b^ Department of Biology, University of Padova, Via U. Bassi 58/b, 35121, Padova, Italy

^c^ Department of Agronomy Food Natural Resources Animals and Environment, University of Padova, Viale dell’Universitá, 16, 35020 Legnaro (PD), Italy

^d^ Department of General Biology, Federal University of Viçosa, Av. Peter Henry Rolfs, s/n, Campus Universitário, 36570-900, Viçosa, Minas Gerais, Brazil

^e^ Department of Animal Science, Universidade Federal de Viçosa, Viçosa, Brazil

^f^ CRIBI Biotechnology Center Viale G. Colombo 3, 35121 Padova, Italy

*Address correspondence to Stefano Campanaro, stefano.campanaro@unipd.it

Supplementary Table S1. Antibiotic disk and its code/potency used for determining antimicrobial resistance.

| **Antibiotic (potency)** | ***S. aureus* 3059**  **(diameter in mm)** | ***S. aureus* UFV2030RH1**  **(diameter in mm)** | **Resistance breakpoint**  **(diameter in mm)** | **Susceptibility breakpoint**  **(diameter in mm)** |
| --- | --- | --- | --- | --- |
| Ampicillin (AMP 10) | 37 | 38 | <16 | >22 |
| Azithromycin (AZI 15) | 31 | 28 | <13 | >18 |
| Cefoxitin (CFO 30) | 28 | 28 |  |  |
| Ciprofloxacin (CIP 05) | 35 | 30 | <21 | >22 |
| Clindamycin (CLI 02) | 29 | 26 | <15 | >21 |
| Chloramphenicol (CLO 30) | 26 | 25 | <14 | >21 |
| Erythromycin (ERI 15) | 30 | 28 | <12 | >18 |
| Gentamicin (GEN 10) | 27 | 23 | <13 | >23 |
| Linezolid (LNZ 30) | 22 | 31 | <12 | >15 |
| Oxacillin (OXA 01) | 23 | 27 | <20 | >21 |
| Penicillin G (PEN 10) | 40 | 39 | - | - |
| Rifampin (RIF 05) | 36 | 33 | <28 | >29 |
| Trimethoprim sulfa (SUT 25) | 30 | 32 | <16 | >20 |
| Tetracycline (TET 30) | 24 | 26 | <10 | >16 |
| Vancomycin (VAN 30) | 26 | 20 | <14 | >19 |
| Amoxicillin/clavulanic acid (AMC 30) | 40 | 42 | <14 | >17 |
| Amikacin (AMI 30) | 33 | 18 | <13 | >18 |
| Ceftriaxone (CRO 30) | 30 | 26 | <14 | >17 |
| Cephalothin (CFL 30) | 40 | 41 | <19 | >23 |
| Amoxicillin (AMO 10) | 40 | 33 | <14 | >18 |
| Cefepime (CPM 30) | 32 | 28 | <13 | >18 |
| Aztreonam (ATM 30) | 6 | 6 | <18 | >25 |
| Piperacillin/tazobactam (PIT 110) | 40 | 35 | <17 | >21 |
| Ceftazidime (CAZ 30) | 28 | 25 | <17 | >21 |
| Bacitracin (BAC 10) | 11 | 6 | <17 | >21 |

Supplementary Table S2. Genbank accession numbers of 27 S. aureus genomes used for the comparative analysis.

| **Strain** | **Accession number** |
| --- | --- |
| *Staphylococcus aureus* subsp. *aureus* 11819-97 | CP003194 |
| *Staphylococcus aureus* strain Mw2 | CP026073 |
| *Staphylococcus aureus* strain 1269 | LNOO01000001 |
| *Staphylococcus aureus* subsp. *aureus* ED133 | CP001996 |
| *Staphylococcus aureus* RF122 | NC_007622 |
| *Staphylococcus aureus* 3059 | CP039848 |
| *Staphylococcus aureus* strain 302 | LNOR01000001 |
| *Staphylococcus aureus* strain 170 | LNOQ01000001 |
| *Staphylococcus aureus* strain NCTC10344 | LS483324 |
| *Staphylococcus aureus* subsp. *aureus* strain ATCC 6538 | CP020020 |
| *Staphylococcus aureus* UFV2030RH1 | SSWQ00000000 |
| *Staphylococcus aureus* subsp. *aureus* str. Newman DNA | AP009351 |
| *Staphylococcus aureus* subsp. *aureus* VC40 | CP003033 |
| *Staphylococcus aureus* subsp. *aureus* COL | CP000046 |
| *Staphylococcus aureus* subsp. *aureus* USA300_FPR3757 | CP000255 |
| *Staphylococcus aureus* subsp. *aureus* USA300_TCH1516 | CP000730 |
| *Staphylococcus aureus* subsp. *aureus* JKD6159 | CP002114 |
| *Staphylococcus aureus* subsp. *aureus* strain MRSA252 | BX571856 |
| *Staphylococcus aureus* strain Bmb9393 | CP005288 |
| *Staphylococcus aureus* subsp. *aureus* TW20 | FN433596 |
| *Staphylococcus aureus* RF122 | NC_007622 |
| *Staphylococcus aureus* subsp. *aureus* Mu50 | BA000017 |
| *Staphylococcus aureus* subsp. *aureus* N315 | BA000018 |
| *Staphylococcus aureus* subsp. *aureus* JH1 | CP000736 |
| *Staphylococcus aureus* subsp. *aureus* CN1 | CP003979 |
| *Staphylococcus aureus* subsp. *aureus* NCTC 8325 | CP000253 |
| *Staphylococcus aureus* subsp. *aureus* ST398 | AM990992 |
| *Staphylococcus aureus* subsp. *aureus* JH9 | CP000703 |
| Staphylococcus argenteus MSHR1132 | FR821777 |
| *Staphylococcus aureus* strain 1364 | LNOP01000001 |
